# Poison and Poisoners

**Published:** 1891-06-20

**Authors:** 


### Poisons and Poisoners.

### III.—ACONITIA: DR. LAMSON.

It is not yet ten years since George Henry Lamson was condemned and executed for the murder of his brother-in-law, Percy John, and the public memory probably still retains some general notion of the crime; but it is worth while to narrate it over again because of two things—the difficulty of proving the presence of aconitia, the poison alleged to have been used, and the prisoner's mental condition.

Percy John was nineteen years of age at the time of his death, and was a pupil—probably a rather privileged one—at a school at Wimbledon. He was an invalid, suffering from curvature of the spine, which threatened to be ultimately, but not immediately fatal to him. If he lived to be twenty-one he would come into possession of £3 000, which, in the event of his dying before that age, would be divided between his two sisters, Mrs. Chapman and Mrs. Lamson. As by the terms of the latter's marriage settlement any property of her's fell into her husband's possession, Dr. Lamson would have benefited by Percy John's death to the extent of £1,500. Add to this that Lamson was in straitened circumstances, and it may be inferred that he had a motive for desiring his brother-in law's death. Still, to desire a man's death is not equivalent to taking his life, and impecunious people do not set about to kill their rich relations, even when they are most convinced that the latter's shoes would be most becoming and comfortable to their own feet. Had this been all that could be proved against Lamson he would certainly never have been condemned.

Unfortunately for him, however, it seemed that this was but the final, and ultimately successful attempt of three, he had made upon the poor lad's life. In August of the same year (1881) in which Percy John was murdered, Lamson visited him at Shanklin, where he was then staying with his sister, Mrs. Chapman, and gave him a pill, which caused symptoms similar to those which marked his fatal illness. Dr. Lamson was then about to sail for America, whence he sent a box of pills to Mr. Bedbrook, at whose school John was placed, saying that "he had met some one in America suffering from the same complaint as the boy, who had derived great benefit from taking medicine similar to that now sent, and requesting Mr. Bedbrook to see the boy take the medicine." One of these, however, made the patient unwell, and he refused to take more of them. It is obvious that in his dual capacity of doctor and relative, Lamson had unequalled opportunities for administering drugs of any sort.

In October he returned from America. The state of his finances may be gathered from the fact that a cheque which he got a tradesman to cash for him was subsequently dishonoured at the bank. Yet on December 1st we find him preparing to go to Paris. On the following day he went to Wimbledon, accompanied by a friend of his, a medical student named Tulloch. They parted there, Tulloch waiting for Lamson in a public-house. When the latter returned, after an absence of about twenty minutes, he said that he had seen his brother-in-law, who was very much worse, and probably would not live long. But when he made this statement, he had not been at the school at all; and it was not till the following evening, that of December 3rd, that he appeared there, with some small gifts for Percy John—cake, sweets, and a box of gelatine capsules, intended to facilitate the swallowing of nauseous medicines. Mr. Bedbrook, the schoolmaster, was present at the interview between the brothers-in-law, and offered Lamson wine. The latter took some, and then asked for some powdered sugar "to cure the effect of the alcohol in the wine." He then produced the capsules, gave an empty one to Mr. Bedbrook, and, having filled another with the sugar, handed it to John, saying, "Here, Percy, you are a swell pill-taker, take this and show Mr. Bedbrook how easy it is to swallow." Percy did so, and soon after Lamson left the school. Within twenty minutes the boy complained of heartburn, which soon developed into a severe attack of sickness, accompanied by great pain in the stomach, constriction of the throat, and uncontrollable restlessness. A doctor was called, whose injections of morphia gave temporary relief, but no real improvement ensued, and in four hours Percy John was dead.

The circumstances of his death were such as to demand a post-mortem examination and the analysis of the internal organs of the body. From this analysis Drs. Stevenson and Dupré, who conducted it, inferred that death was due to poisoning by aconitia. They surmised this from the effect produced upon themselves by tasting small quantities of the substances handed to them, and also from the fact that injections of extracts made from them produced the same effect on mice as injections of pure aconitia. Mr. Montague Williams, who defended the prisoner with marked ability, contended that experiments on animals could not be regarded as conclusive, and that hypotheses based mainly on the sense of taste were, in the highest degree, unsatisfactory. Mr. Williams also tried to prove that the alkaloids found in the body—due, the medical experts held, to the presence of aconitia—might be the "cadaveric alkaloids" which are spontaneously produced in the process of putrefaction. The *post mortem* examination had, however, been carried out too soon to permit of these cadaveric alkaloids being evolved, nor do they ever under analysis give the same results as aconite. Moreover, there was against Lamson the serious fact that in the end of November he had tried to buy aconitia at one chemist's, and being advised by a cautious attendant to "go where he was better known," had made his purchase at an establishment where the salesmen were more easy-going. Taking into consideration this buying of the poison, Percy John's previous illness after receiving medicine from him, his prediction that the boy would not live long while as yet there was no foundation for the statement, and the reason his circumstances gave him for desiring his brother-in-law's death, it was little wonder that Lamson was condemned.

After the verdict, indeed, his own friends seemed to give up saying that he had not poisoned Percy John, and sought a commutation of his sentence on the plea that he was not responsible for his actions. There was insanity in his family. His grandmother and her brother had died in an asylum, as also had an aunt. True, the two first had suffered at an advanced age from senile dementia, while the madness of the third was a result of puerperal fever, and no evidence of the mental condition of nearer relatives was offered. But Lamson's own record spoke of a mind diseased, though not, perhaps, unhinged. As a medical student he had been wild, erratic, and gloomy, fond of "chemical experiments of a morbid character," and given to administering medicines with absolute recklessness as to quantity and results. In 1877 he had acted as surgeon during the Servian war, and had then, it was said, shown a mania for administering aconitia in every case. Further, he had fallen a victim to the morphia habit, and had shown all the moral and intellectual results that follow enslavement to that deadly drug. There seems, indeed, no doubt that Lamson's was "a mind diseased," and in any other country in Europe it is probable that he would have escaped punishment. But in England, though the pathology of crime is not deeply studied, there is a common-sense in the popular mind that probably answers better for practical purposes; and that common-sense, in the person of Sir William Harcourt, then Home Secretary, refused to accept this plea of insanity. Had Lamson been insane, he would not in all likelihood have chosen as his victim the one person in the world whose death was sure to benefit him in money matters. There was a method in his madness. If we regard all sin as aberration we may raise the plea of lunacy for him; but so long as we admit the existence of conscience and will, we can accept such a plea only when a crime seems utterly motiveless or due to some motive of the least mundane kind, among which we cannot by any means reckon the need or the desire for gold. Lamson's lunacy was such as is common to all who let selfish desires overmaster them, and however demoralised his constitution might be by opium, he was not under the influence of that narcotic when he committed the crime; and in choosing a poison whose action was little known, and which was difficult to trace and test, he manifested, not insanity, but very marked acuteness. Doubtless, he was often on the borderland between sense and madness, but at the time he contemplated and committed the murder he was sane enough. The delirium of drunkenness or the irresponsibility of madness may be regarded as extenuating a crime by showing that it was not premeditated; but justice would become a farce if the fact that a person had contracted a vicious habit were held to condone all his evil deeds. If there were no vices in the world there would be no crimes, for no man whose moral nature was perfectly balanced would ever wrong his neighbour. At the same time it is not wise to treat the wanton selfishness of cruelty as if it were unconscious and irresponsible. A time may, indeed, come when we shall be justified in assuming that every sin is the outcome of disease, and treating it accordingly. But the world is not yet perfect.

